# The relationship between perceived stress and depression in colorectal cancer patients: the mediating role of illness perception and the moderating role of self-efficacy

**DOI:** 10.3389/fonc.2026.1746202

**Published:** 2026-02-18

**Authors:** Fuzhuo Wang, Jiashuang Xu, Hong Sun, Xiuli Wang, Zhongguang Che, Ye Huang

**Affiliations:** 1 The First Affiliated Hospital of Jinzhou Medical University, Jinzhou, Liaoning, China; 2 Nursing Department, Peking Union Medical College Hospital, Chinese Academy of Medical Sciences & Peking Union Medical College, Beijing, China; 3 Liaoning Cancer Hospital & Institute, Shenyang, Liaoning, China

**Keywords:** colorectal cancer, depression, illness perception, perceived stress, self-efficacy

## Abstract

**Background:**

Numerous studies have demonstrated a close association between perceived stress and depression in colorectal cancer patients; however, the underlying mechanisms remain incompletely understood. This study aims to investigate the impact of perceived stress on depression in this population, as well as the mediating role of illness perception and the moderating role of self-efficacy.

**Method:**

A cross-sectional design was employed. From May to November 2024, a questionnaire survey was conducted among 290 colorectal cancer patients at two Grade A tertiary hospitals in Shenyang and Jinzhou, Liaoning Province, China. The questionnaire comprised sections on general demographics, perceived stress, illness perception, self-efficacy, and depression. Descriptive statistics and correlation analyses were performed using SPSS 25.0 and the PROCESS 3.5 macro. Mediation and moderation effects were tested using bootstrap resampling.

**Results:**

A significant positive correlation was found between perceived stress and depression (*β* = 0.483, *P* < 0.001) and this relationship was partially mediated by illness perception (*β* = 0.083). Self-efficacy moderated the association between perceived stress and illness perception (*β* = 0.024, *P* < 0.001), with higher levels of self-efficacy strengthening the relationship between perceived stress and illness perception.

**Conclusion:**

This study identifies illness perception as a mediating pathway in the association between perceived stress and depression, while self-efficacy moderates the relationship between perceived stress and illness perception. Accordingly, a multidimensional clinical approach may be considered for addressing depressive symptoms in colorectal cancer patients. Such an approach could concurrently target perceived stress reduction, modification of illness perception, and enhancing self-efficacy.

## Introduction

Colorectal cancer, one of the most prevalent malignant tumors worldwide, imposes not only physical burdens but also significantly impacts patients’ mental health throughout diagnosis and treatment (1, 2). Depression, the most common psychological disorder among colorectal cancer patients (3), leads to symptoms such as low mood, loss of interest, sleep disturbances, and decreased appetite (4). Depression in these patients is associated with poorer treatment outcomes and prognosis, and constitutes a significant risk factor for suicidal ideation and behavior (5, 6). Therefore, investigating the factors influencing depression and their underlying mechanisms in colorectal cancer patients is clinically important for developing targeted psychological interventions, improving psychological well-being, and enhancing prognosis.

Perceived stress refers to an individual’s subjective experience resulting from the appraisal of environmental stressors, reflecting a perceived imbalance between situational demands and coping capacity (7). Substantial evidence confirms that perceived stress is prevalent among cancer patients and serves as a strong predictor of depression (8, 9). This association has been consistently observed in colorectal cancer populations across both cross-sectional and longitudinal studies (10–12). Despite this established link, the psychological mechanisms remain poorly understood.

Illness perception, defined as the cognitive representation of illness shaped by personal experience and knowledge (13), is a key factor influencing self-management, emotional adjustment, and clinical outcomes (14). Negative illness perception is closely associated with greater psychological distress, including anxiety and depression (15–17). Furthermore, perceived stress has been identified as a significant antecedent of negative illness perception (18, 19). To theoretically construct this relationship, we draw on Leventhal’s Common-Sense Model of Self-Regulation (CSM) (20). The CSM is a dynamic framework explaining how individuals cope with health threats. Its core components include: cognitive representation (the individual’s understanding of the illness’s identity, cause, timeline, consequences, and controllability), emotional representation (emotional responses triggered by illness cognition), and a coping process involving primary appraisal (threat judgment) and secondary appraisal (coping resource evaluation). In this context, perceived stress among colorectal cancer patients can be viewed as the outcome of primary appraisal within the CSM. Through secondary appraisal, which integrates personal experience and medical information, patients are prone to form negative illness perception (e.g., perceiving severe consequences, a long timeline, and low controllability of the illness). These perception tendencies are, in turn, associated with depression. Therefore, from the CSM theoretical perspective, illness perception constitutes a plausible cognitive pathway through which perceived stress influences depression.

Meanwhile, according to Bandura’s Social Cognitive Theory (21), self-efficacy (defined as an individual’s belief in their ability to cope) (19) is considered a key moderating variable in cognitive processing under stress. A central tenet of this theory posits that self-efficacy actively shapes the cognitive adaptation process by influencing the individual’s appraisal frame and interpretative style toward stressful events. Specifically, high self-efficacy may prompt individuals to appraise perceived stress as a “manageable challenge,” thereby initiating a series of adaptive cognitive processes (such as actively seeking information and engaging in rational reappraisal). This proactive, in-depth processing is expected to strengthen the association between perceived stress and illness perception (22). Conversely, low self-efficacy tends to lead to an appraisal of “uncontrollable threat,” which can trigger catastrophizing interpretations, thereby weakening the link between perceived stress and illness perception (23). Hence, this study proposes that self-efficacy may play a positive moderating role in the relationship between perceived stress and illness perception; that is, higher levels of self-efficacy are likely to strengthen the effect of perceived stress on illness perception.

**Figure 1:**
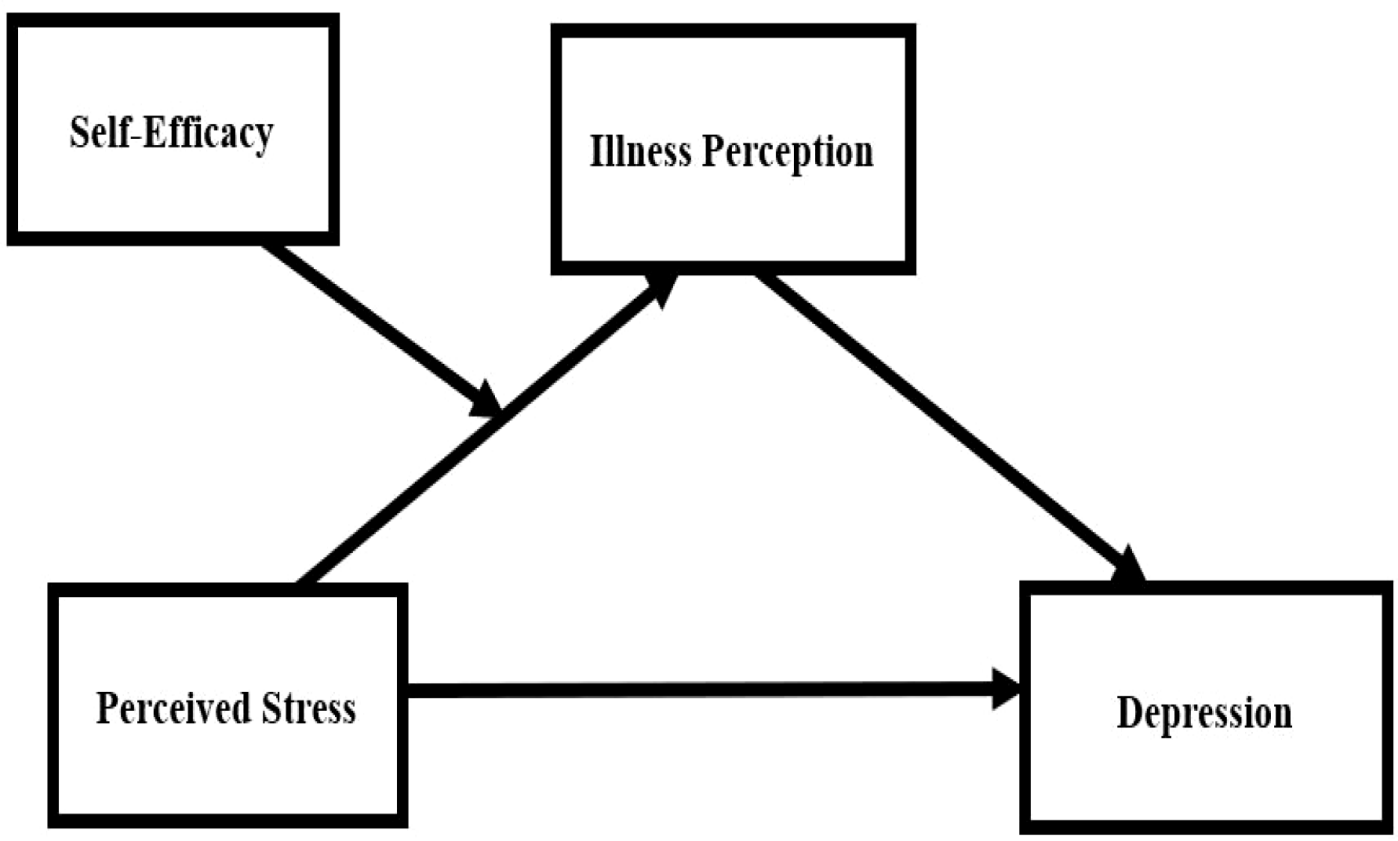
The hypothesized moderated mediation model.

In summary, this study innovatively integrates the Common-Sense Model of Self-Regulation and Social Cognitive Theory to construct a moderated mediation model (**Figure 1**), aiming to systematically examine the following two hypotheses: (1) Illness perception mediates the relationship between perceived stress and depression; and (2) Self-efficacy positively moderates the pathway from perceived stress to illness perception. The implementation of this research is expected to provide a novel perspective for uncovering the psychological mechanisms underlying depressive symptoms in colorectal cancer patients, as well as a theoretical basis for developing multidimensional intervention programs that integrate stress management, cognitive restructuring, and efficacy enhancement.

## Methods

### Study design and setting

This study employed a cross-sectional design. Data collection took place from May to November 2024 at two Grade-A tertiary hospitals in Liaoning Province, China.

### Participants and sampling

A multistage stratified random sampling method was used. Inclusion criteria were: (1) diagnosis of colorectal cancer confirmed by clinical pathology; (2) voluntary provision of informed consent; (3) clear consciousness and adequate communication ability; (4) age ≥18 years. Exclusion criteria were: (1) critically ill patients unable to complete the process; (2) clinically assessed life expectancy of less than 6 months; (3) presence of cognitive impairment or diagnosed psychiatric disorders; (4) history of psychological interventions that could interfere with study outcomes; (5) individuals unaware of their diagnosis.

### Sampling procedure

The sampling procedure consisted of three stages. Stage 1: Within Liaoning Province, eight Grade III Class A hospitals with specialized oncology departments were selected based on national rankings. Using hospital primary specialty as the stratification variable and a random number table, Dalian University of Technology Affiliated Cancer Hospital and Jinzhou Medical University Affiliated Hospital were selected (sampling proportion 25%). Stage 2: Within each hospital, departments admitting colorectal cancer patients were listed and stratified by primary treatment modality. Using random number tables, selected departments were: Dalian University of Technology Affiliated Cancer Hospital: Colorectal Surgery Wards 5 and 6, Department of Integrated Traditional and Western Medicine; Jinzhou Medical University Affiliated Hospital: General Surgery Colorectal Wards 1 and 3, Oncology Department. Stage 3: Researchers visited selected departments weekly. Eligible patients were screened from the daily inpatient lists. Systematic random sampling was applied: eligible patients were listed by bed number, a random starting point was determined, and every other patient (k=2) was invited. This ensured approximately 50% systematic sampling of daily eligible patients, enabling efficient recruitment while maintaining randomness.

### Sample size estimation

Sample size was determined using a dual approach. First, based on the factor analysis guideline of 10–20 participants per scale dimension, and considering the 7 dimensions across the scales used in this study, a preliminary sample size estimate ranged from 70 to 140 (24). Second, an *a priori* power analysis was conducted using G*Power 3.1.9.4 software. Specifically, we selected the statistical test “F-test—Linear Multiple Regression: Fixed Model, R² Deviates from Zero.” The analysis targeted the hypothesized moderated mediation model, which included 12 predictor variables (8 demographic variables, perceived stress, illness perception, self-efficacy, and their interaction term). Parameters were set as α = 0.05 (two-tailed), statistical power = 0.95, and a medium effect size (f² = 0.15) (25). This calculation yielded a minimum required sample size of 221. Accounting for an estimated 20% rate of invalid questionnaires or attrition, the target sample size was adjusted to 277 [221/(1-0.20)]. In practice, to ensure data quality and representation, the scope of recruitment was expanded. A total of 319 questionnaires were distributed, and 290 valid questionnaires were ultimately retained (159 from Dalian University of Technology Affiliated Cancer Hospital and 131 from Jinzhou Medical University Affiliated Hospital), resulting in a valid response rate of 90.91%.

### Data collection procedure

Before data collection, all research personnel completed a standardized training program (26–28). Standardized training refers to structured, uniform training procedures implemented for all research personnel to ensure the consistency, accuracy, and reliability of research data. The program consisted of two key modules: (1) scoring criteria and tool familiarization to ensure consistent understanding of the measurement tools (Perceived Stress Scale, Self-Efficacy Scale, Illness Perception Scale, and Depression Scale) and their scoring protocols; and (2) patient-centered communication skills, focusing on techniques for building rapport, delivering clear and neutral instructions, and creating a supportive environment to facilitate honest and accurate responses.

### Ethical considerations

This study was approved by the Research Ethics Committee of Jinzhou Medical University (Approval No. JZMULL2023029). All procedures were in accordance with the ethical standards of the institutional committee and the Declaration of Helsinki. Written informed consent was obtained from all participants.

### Instruments

#### General demographic characteristics questionnaire

This questionnaire followed commonly used standards in psychosocial research (29), covering age, gender, education, marital status, economic status, residence, hospitalization count, and tumor staging. Categories were defined as follows: Age (≤44, 45-59, ≥60 years); Gender (male, female); Education (elementary school or below, junior high school, high school/vocational school, college or above); Marital status (unmarried, married, divorced, widowed); Economic status (below means, balanced, surplus); Residence (rural, urban); Hospitalization count (1, 2, 3, ≥4 times); Tumor stage (I, II, III, IV).

#### Perceived stress scale

The 14-item Perceived Stress Scale by Cohen et al. (1983) (30) comprises two dimensions: tension and helplessness. Items are rated on a 5-point Likert scale (0=“Never” to 4=“Very much”). Seven items are reverse-scored. Total scores range from 0-56, categorized as mild (0-28), moderate (29-42), or excessive (43-56) perceived stress. In this study, Cronbach’s α was 0.814, and KMO = 0.648.

#### Self-efficacy scale

The 10-item General Self-Efficacy Scale by Schwarzer et al. (1992) (31) uses a 5-point Likert scale (1=“Disagree” to 5=“Strongly Agree”). Total scores range from 10-50, categorized as low (<25), moderate (25-37), or high (>37) self-efficacy. In this study, Cronbach’s α was 0.935, KMO = 0.936.

#### Illness perception scale

The Brief Illness Perception Questionnaire by Broadbent et al. (2006) (32) consists of 9 items across three dimensions: disease cognition, emotion, and comprehension. Item 9 is open-ended and not scored. The first 8 items are rated on a 0–10 scale (three reverse-scored), yielding a total score of 0-80. Higher scores indicate stronger negative perceptions. In this study, Cronbach’s α was 0.752, KMO = 0.713.

#### Depression scale

The 9-item Patient Health Questionnaire (PHQ-9) by Kroenke et al. (2001) (33) uses a 4-point scale (0=“Not at all” to 3=“Nearly every day”). Total scores range from 0-27, categorized as: 0-4 (none), 5-9 (mild), 10-14 (moderate), 15-19 (moderately severe), 20-27 (severe). In this study, Cronbach’s α was 0.875, KMO = 0.891.

### Statistical analysis

Data analysis was conducted using IBM SPSS Statistics 25.0 and the PROCESS macro version 3.5. Normality of continuous variables was assessed via the Kolmogorov-Smirnov test; all variables were normally distributed (*P* > 0.05). Descriptive statistics included frequencies/percentages for categorical variables and mean ± standard deviation for continuous variables. Independent samples t-tests and one-way ANOVA were used to examine differences in depression scores across demographics. Pearson correlation analysis evaluated associations among study variables. Moderated mediation analysis was performed using PROCESS macro 3.5 (Model 14 for moderation, Model 4 for mediation). Effects were examined using bootstrap resampling (5,000 samples) to generate 95% bias-corrected confidence intervals (CI). A two-tailed *P* < 0.05 was considered statistically significant.

## Results

### Descriptive statistics

**Table 1** presents demographic characteristics and univariate analysis. Participants’ mean age was 59.58 ± 13.09 years (range 19-90). The sample comprised 149 males (51.4%) and 141 females (48.6%). Most were married (81.0%). Univariate analysis revealed significant associations between depression and age, gender, education level, marital status, hospitalization count, and pathological stage (*P* < 0.05). These variables were retained as covariates in subsequent analyses.

**Table 1:** Univariate analysis of depression in colorectal cancer patients based on different characteristics (N = 290).

Variables	Group	*N*(%)	*M ±* SD	*F/t*	*P*
Age	≤44	42 (14.5)	6.24 ± 4.08	2.306	0.001
	45-59	84 (29.0)	8.57 ± 4.86		
	≥60	164 (56.6)	10.26 ± 4.17		
Gender	Male	149 (51.40)	10.07 ± 4.80	15.308	<0.001
	Female	141 (48.60)	8.26 ± 4.15		
Educational level	Elementary school and below	144 (49.7)	10.37 ± 4.36	10.722	<0.001
	Junior high school	77 (26.6)	9.00 ± 4.63		
	High school or vocational school	33 (11.4)	7.88 ± 3.29		
	College or above	36 (12.4)	6.06 ± 4.65		
Marital status	Unmarried	13 (4.5)	6.15 ± 5.06	3.955	0.009
	Married	235 (81.0)	9.06 ± 4.42		
	Divorced	8 (2.8)	11.13 ± 5.30		
	Widowed	34 (11.7)	10.79 ± 4.75		
Economic Status	Income falls short of expenses	176 (60.7)	9.27 ± 4.51	0.106	0.900
	Income and expenses are balanced	85 (29.3)	9.13 ± 4.48		
	Income exceeds expenses	29 (10)	8.86 ± 5.39		
Residence	Rural	81 (27.9)	9.07 ± 4.85	0.067	0.796
	Urban	209 (72.1)	9.23 ± 4.48		
Hospitalization Count	1 time	144 (29.7)	8.42 ± 3.42	0.067	0.796
	2 times	102 (35.2)	10.83 ± 4.99		
	3 times	26 (9.0)	6.85 ± 5.53		
	≥4 times	18 (6.2)	9.33 ± 6.02		
Pathological Stage	I	38 (13.1)	7.45 ± 5.76	10.313	<0.001
	II	122 (42.1)	8.38 ± 4.13		
	III	86 (29.7)	9.59 ± 4.15		
	IV	44 (15.2)	12.14 ± 4.08		

### Correlations between variables

As shown in **Table 2**, perceived stress was negatively correlated with self-efficacy and positively correlated with illness perception and depression (*P* < 0.01). Self-efficacy was negatively correlated with illness perception and depression (*P* < 0.01). Illness perception was positively correlated with depression (*P* < 0.01). Mean scores were: perceived stress 29.56 ± 6.45, self-efficacy 32.20 ± 7.15, illness perception 29.33 ± 4.63, depression 9.19 ± 4.58.

**Table 2:** Correlation analysis.

Variables	*M* ± SD	1	2	3	4
Perceived stress	29.56 ± 6.45	1			
Self-efficacy	32.20 ± 7.15	-0.617^**^	1		
Illness perception	29.33 ± 4.63	0.556^**^	-0.456^**^	1	
Depression	9.19 ± 4.58	0.713^**^	-0.597^**^	0.520^**^	1

^∗∗^*P* < 0.01.

### Mediation analysis

As shown in **Table 3**, after controlling for covariates, mediation analysis (PROCESS Model 4) indicated a significant positive correlation between perceived stress and depression (*β* = 0.483, *P* < 0.001). When illness perception was introduced as a mediator, the direct effect remained significant (*β* = 0.400, *P* < 0.001). Perceived stress was positively correlated with illness perception (*β* = 0.515, *P* < 0.001), and illness perception was positively correlated with depression (*β* = 0.161, *P* < 0.001). The indirect effect was significant (*B* = 0.083, SE = 0.026, 95% CI = [0.036, 0.137]), indicating partial mediation. The mediating effect accounted for 17.18% of the total effect (**Table 4**).

**Table 3:** Mediation analysis.

Predictors	Depression				Perceived stress				Depression			
	*B*	SE	*t*	95%CI	*B*	SE	*t*	95%CI	*B*	SE	*t*	95%CI
Age	-0.576	0.349	-1.648	(-1.265, 0.112)	1.693	0.532	3.182^**^	(0.646, 2.741)	-0.849	0.346	-2.457^*^	(-1.530, -0.169)
Gender	-0.437	0.393	-1.112	(-1.211,0.337)	2.501	0.598	4.182^***^	(1.324, 3.678)	-0.840	0.393	-2.136^*^	(-1.615, -0.069)
Educational level	-0.956	0.229	-4.181^***^	(-1.406, -0.506)	-0.083	0.348	-0.239	(-0.768, 0.602)	-0.943	0.222	-4.244^***^	(-1.379, -0.505)
Marital status	0.108	0.281	0.386	(-0.444, 0.661)	0.014	0.427	0.033	(-0.826, 0.855)	0.106	0.273	0.389	(-0.431, 0.643)
Hospitalization Count	0.681	0.224	3.034^**^	(0.239, 1.122)	1.301	0.341	3.813^***^	(0.629, 1.973)	0.471	0.223	2.107^*^	(0.031, 0.910)
Pathological staging	0.201	0.219	0.917	(-0.23, 0.631)	1.218	0.333	3.659^***^	(0.563, 1.873)	0.004	0.217	0.019	(-0.424,0.432)
Perceived stress	0.483	0.032	14.887^***^	(0.419,0.547)	0.515	0.049	10.423^***^	(0.417, 0.612)	0.400	0.037	10.790^***^	(0.327, 0.473)
Illness perception									0.161	0.038	4.243^***^	(0.087, 0.236)
*R*	0.746				0.685				0.763			
*R^2^*	0.556				0.469				0.583			
*F*	50.428^***^				35.569^***^				49.03^***^			

^*^P < 0.05, ^**^P < 0.01, ^***^P< 0.001.

**Table 4:** Tests for mediating effect.

Effect	Effect value	SE	LLCI	ULCI	Effect ratio %
Total effect	0.483	0.033	0.419	0.547	100
Direct effect					
X→Y	0.400	0.037	0.327	0.535	82.82
Indirect effect					
X→M→Y	0.083	0.026	0.036	0.137	17.18

X, is Perceived stress; M, is Illness perception; Y, is Depression.

### Moderated mediation analysis

Moderated mediation analysis (PROCESS Model 14) was conducted, controlling for covariates. As shown in **Table 5**, the interaction term between perceived stress and self-efficacy was positively correlated with illness perception (*B* = 0.024, *P* < 0.001), indicating that self-efficacy moderated the “perceived stress → illness perception” pathway.

**Table 5:** Moderated mediation analysis.

Variables	Illness perception				Depression			
	*B*	SE	*t*	95%CI	*B*	SE	*t*	95%CI
Age	1.398	0.519	2.693^***^	(0.376, 2.420)	-0.849	0.346	-2.457^*^	(-1.530, -0.169)
Gender	2.413	0.579	4.170^**^	(1.274, 3.552)	-0.840	0.393	-2.136^*^	(-1.614, -0.066)
Educational level	-0.199	0.337	-0.589	(-0.862, 0.465)	-0.943	0.222	-4.244^***^	(-1.379, -0.506)
Marital status	-0.161	0.414	-0.389	(-0.977, 0.654)	0.106	0.273	0.389	(-0.431, 0.643)
Hospitalization Count	1.411	0.337	4.191^***^	(0.748, 2.074)	0.471	0.223	2.107^*^	(0.031, 0.910)
Pathological staging	1.014	0.326	3.115^**^	(0.373, 1.655)	0.004	0.217	0.019^*^	(-0.424, 0.432)
Perceived stress	0.404	0.059	6.819^***^	(0.287, 0.520)	0.400	0.037	10.787^***^	(0.327, 0.473)
Self-efficacy	-0.164	0.054	-3.05^**^	(-0.269, -0.058)				
Perceived stress^*^ Self-efficacy	0.024	0.006	4.207^***^	(0.287, 0.520)				
*R*	0.713				0.763			
*R^2^*	0.508				0.583			
*F*	32.125				49.034			

^*^P < 0.05, ^**^P < 0.01, ^** *^P< 0.001.

**Figure 2:**
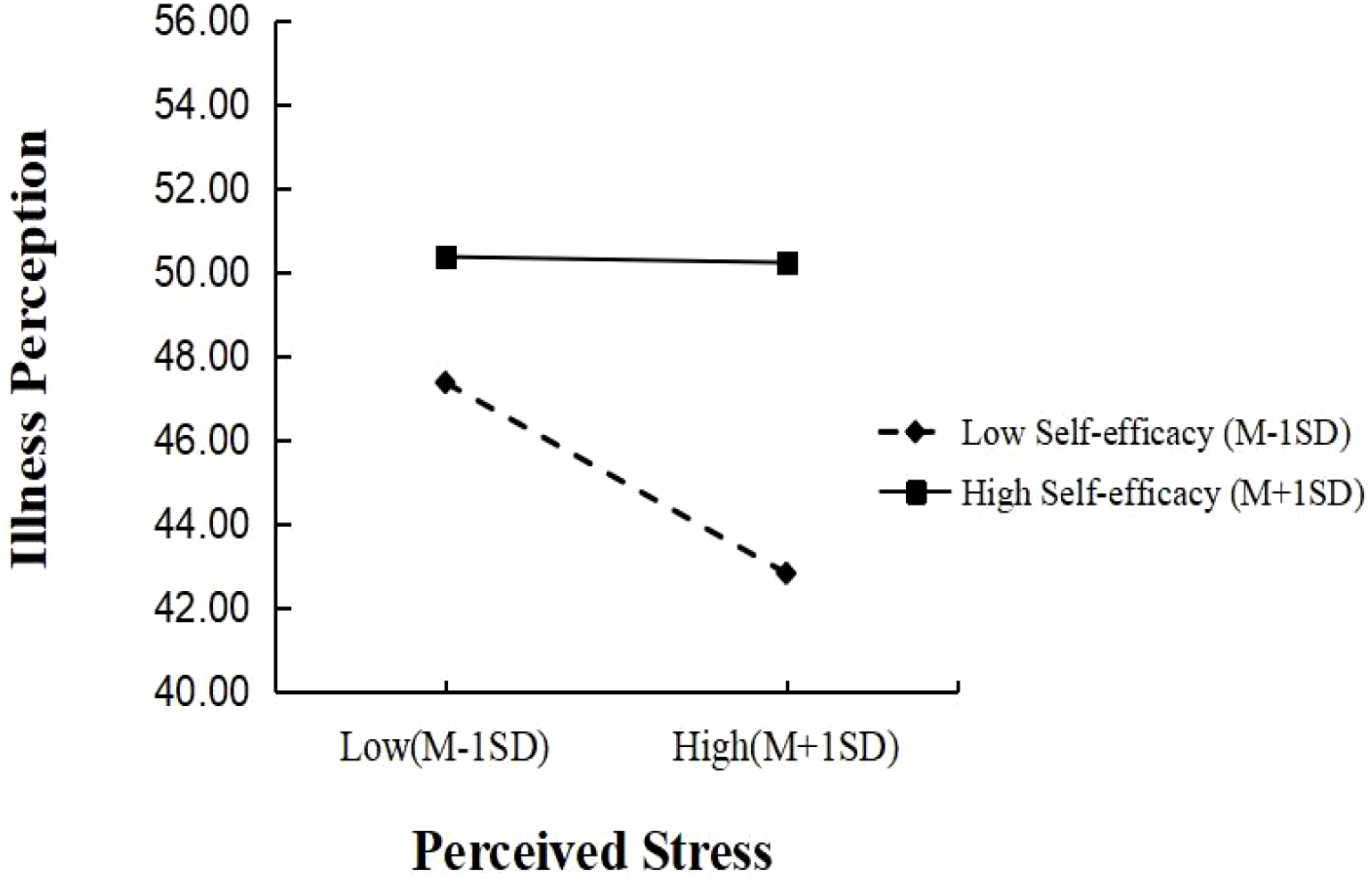
Plot of the relationship between perceived stress and illness perception at two levels of self-efficacy.

To illustrate this moderation, self-efficacy was categorized into three levels (low: *M* - 1SD; medium: *M*; high: *M* + 1SD). Simple slope analysis (**Table 6**; **Figure 2**) showed that among participants with low self-efficacy, perceived stress significantly positively predicted illness perception (*B* = 0.233, SE = 0.077, *t* = 3.026, *P* < 0.01). This positive predictive effect was stronger among participants with high self-efficacy (*B* = 0.575, SE = 0.066, *t* = 8.655, *P* < 0.001).

**Table 6:** Moderating effects of different levels of self-esteem.

Self-efficacy	Effect	SE	LLCI	ULCI
M-1SD	0.233	0.077	0.081	0.384
M	0.403	0.059	0.287	0.520
M+1SD	0.575	0.066	0.444	0.705

### Summary of main findings

**Figure 3:**
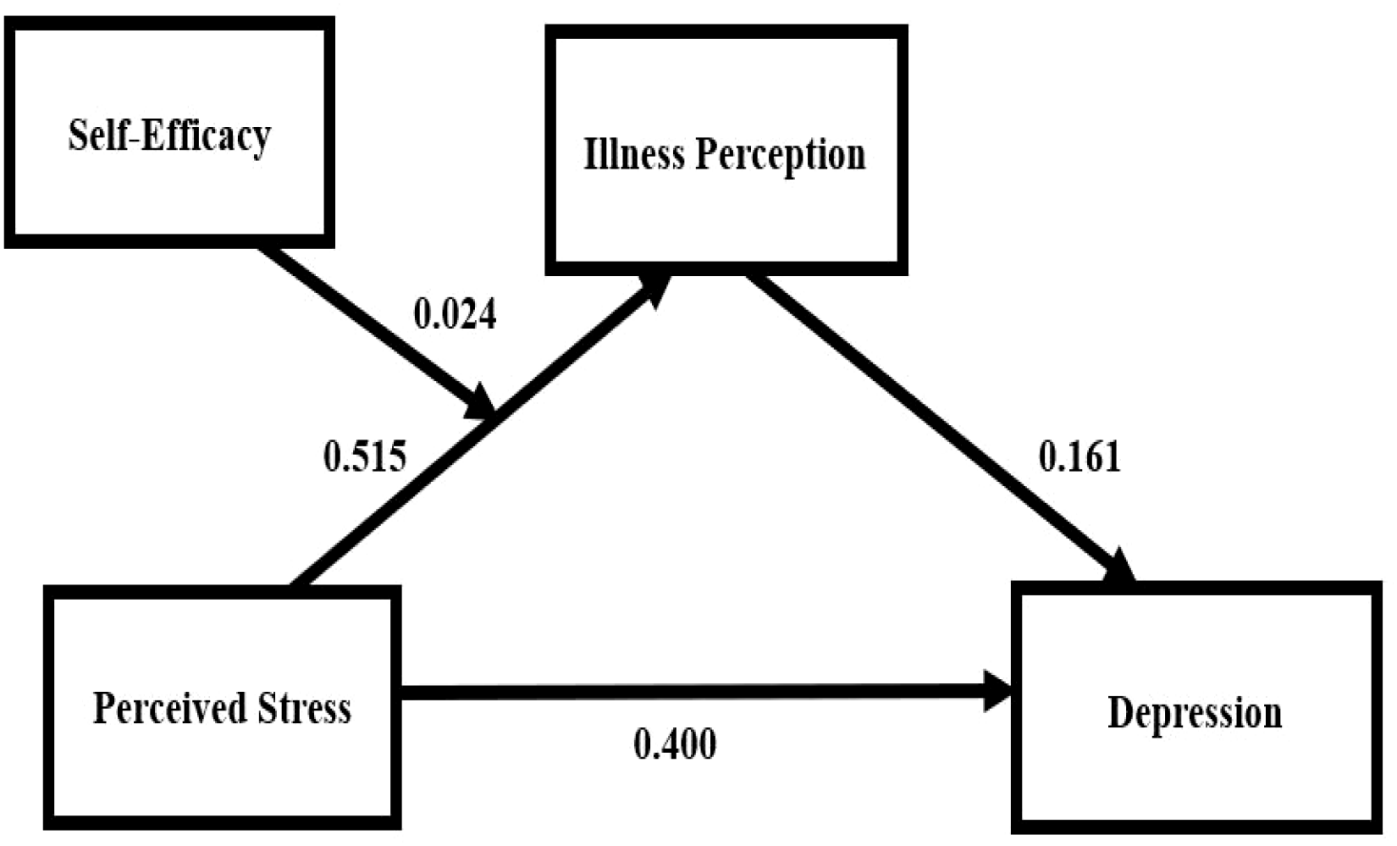
Moderated mediation model.

Overall, perceived stress directly influenced depression and indirectly affected it through illness perception. Self-efficacy moderated the “perceived stress → illness perception” pathway (**Figure 3**).

## Discussion

This study found a significant positive correlation between perceived stress and depression in colorectal cancer patients, consistent with existing literature (34). This relationship may arise from the physical toll of diagnosis and treatments (e.g., surgery, chemotherapy), which deplete patients’ resources and likely intensify perceived stress (35). Mechanistically, from a mechanistic perspective, persistent perceived stress may be associated with depression through dual pathways: physiologically, chronic stress can activate the HPA axis, dysregulate stress hormones (e.g., cortisol), and disturb neurotransmitter balance (e.g., serotonin, dopamine)—processes associated with depression (36). Psychologically, high perceived stress often coincides with feelings of helplessness and drains emotional regulation capacity, hindering adaptive coping with negative affect, which may cumulatively contribute to depression (37). Thus, implementing individualized care focused on reducing perceived stress is recommended as a supportive component of depression prevention and management in this population.

The study also found that illness perception mediates the relationship between perceived stress and depression, consistent with the theoretical framework of Leventhal’s Common-Sense Model of Self-Regulation (20). This model proposes that individuals undergo a cognitive-emotional process of “primary appraisal—secondary appraisal—emotional response” when coping with illness. Colorectal cancer patients face multiple stressors, including diagnostic shock, treatment trauma, recurrence risk, and financial burden (38). These are perceived as stress during primary appraisal, triggering secondary appraisal and forming cognitive representations of disease etiology, progression, and consequences (i.e., illness perception) (39). Negative illness perception may be associated with negative emotions through two pathways: first, by reinforcing the cognitive bias of “stress uncontrollability,” linking to depressive emotional responses; second, by prompting passive coping strategies like avoidance, which diminish the protective effect of social support and may thus correlate with depression (40, 41).

Further analysis indicates that self-efficacy moderates the relationship between perceived stress and illness perception. pathway, and social cognitive theory contributes to a richer understanding of this process (21). Patients with high self-efficacy exhibit greater confidence in their ability to cope with illness, tending to evaluate perceived stress as “manageable challenges.” Consequently, they proactively seek illness information and rationally assess treatment options, facilitating the transformation of stress into clear, controllable illness perception. This results in a stronger association between stress and cognition (42, 43). In contrast, patients with low self-efficacy, lacking confidence in coping, are prone to cognitive avoidance under stress. Stress-related information fails to fully integrate into their illness perception, resulting in a relatively weaker association between the two (44, 45). Consequently, future intervention programs may prioritize enhancing patients’ self-efficacy as a core objective. This can be achieved through setting phased, attainable behavioral goals, providing skill practice, and offering feedback on successful experiences. Such interventions help patients develop more adaptive stress coping and illness perception patterns, potentially yielding positive impacts on their mental health.

### Limitations

This study has several limitations. First, the cross-sectional design can only reveal correlations between variables and cannot infer causal directionality. Future longitudinal or experimental studies may be conducted to further validate the temporal sequence and causal relationships among variables. Second, the sample originates from two hospitals within the same province, potentially limiting the representativeness of the results. Subsequent research may expand the sampling scope to enhance external validity. Additionally, all data were collected through self-reported questionnaires, potentially subject to social desirability and recall bias. Future studies should integrate multi-source data, such as physiological indicators and clinical assessments, to enhance measurement objectivity and strengthen the robustness of conclusions.

### Implication

Based on the moderated mediation model established in this study, a multidimensional psychological support program for colorectal cancer patients should be designed across three levels: First, integrate the Brief Perceived Stress Scale, the Illness Perception Questionnaire, and the General Self-Efficacy Scale into routine care for systematic screening. Patients can be categorized based on assessment results to inform subsequent support program development (46, 47). Second, for patients with elevated perceived stress levels, structured programs such as mindfulness-based stress reduction training and relaxation technique instruction should be offered. Patients with negative illness perception may benefit from visual disease education and cognitive restructuring dialogues to foster more adaptive illness understanding. Those with low self-efficacy can participate in symptom management skill workshops and peer experience-sharing groups, providing platforms for learning and exchange (48). Third, establish a multidisciplinary collaboration mechanism involving healthcare providers and mental health professionals. Integrate digital health tools (e.g., health education apps, mood diary mini-programs) as extensions of in-hospital support. Implement regular follow-up protocols to tailor support content according to patients’ conditions at different treatment stages (49, 50). These comprehensive measures will help build a more holistic support system, thereby playing a positive role in reducing depression risk among colorectal cancer patients.

## Conclusions

Overall, this study indicates that illness perception partially mediates the relationship between perceived stress and depression in colorectal cancer patients, while self-efficacy moderates the association between perceived stress and illness perception. These findings highlight the interconnectedness of perceived stress, illness perception, and self-efficacy with depression in colorectal cancer patients. Therefore, developing targeted interventions may represent a crucial direction for supporting the mental health of colorectal cancer patients in future research and clinical practice.

## Data Availability

The raw data supporting the conclusions of this article will be made available by the authors, without undue reservation.
